# Multimodal imaging of infantile scimitar syndrome with Eisenmenger physiology: A case report of complex intracardiac and extracardiac shunts

**DOI:** 10.1016/j.radcr.2026.06.141

**Published:** 2026-07-20

**Authors:** Berihu Gebre, Yirgalem Gereziher, Catherine Alcober, Gladys Baltazar, Viachaslau Markau

**Affiliations:** a Department or Radiology, Nauru Medical Center, Republic of Nauru; b Department of Pediatric Surgery, Mekelle University, Mekele, Ethiopia; c Department Pediatric, Nauru Medical Center, Republic of Nauru; d Department of Anesthesiology and Intensive Care, Nauru Medical Center, Republic of Nauru

**Keywords:** Scimitar, Eisenmenger, Venolobar syndrome, Atrial septal defect(ASD), Patent ductus arteriosus (PDA)

## Abstract

Scimitar syndrome is a rare congenital vascular malformation in which the right pulmonary veins drain abnormally into the inferior vena cava, right atrium, or portal vein instead of the left atrium. It occurs in about 1-3 per 100,000 live births and has 2 forms: an infantile form, usually more severe and associated with multiple anomalies and poorer prognosis, and a child/adult form, which is often milder or asymptomatic. This report describes an 8-year-old boy diagnosed with complex Scimitar syndrome complicated by Eisenmenger syndrome. Echocardiography, chest X-ray, and computed tomography angiography showed right lung hypoplasia, anomalous drainage of the right pulmonary veins into the supradiaphragmatic inferior vena cava, systemic arterial supply to the right lung, large atrial septal defect and patent ductus arteriosus with bidirectional shunting, severe pulmonary hypertension, and right heart enlargement—findings consistent with long-standing left-to-right shunt and subsequent shunt reversal. Management in Scimitar syndrome depends on shunt size, pulmonary pressures, and associated anomalies. Mild cases with small shunts may be managed with regular follow-up, whereas patients with significant left-to-right shunting, right heart strain, or recurrent infections usually benefit from surgery. In advanced cases with established Eisenmenger physiology, as in this patient, curative surgery is generally not possible and treatment is mainly medical and supportive, underscoring the importance of early imaging-based diagnosis and timely referral.

## Introduction

Scimitar syndrome, also known as congenital venolobar syndrome, was first described as an autopsy finding by the French physician Raoul Chassinat in 1836 [1,2]. It is a rare variant within the group of congenital cardiovascular anomalies known as partial anomalous pulmonary venous return, in which a pulmonary vein drains into the inferior vena cava (IVC), right atrium, or portal vein rather than the left atrium (LA) [2,3]. Scimitar syndrome is characterized by anomalous drainage of the right pulmonary veins, lung sequestration with an aberrant vascular supply, and right lung hypoplasia [4]. In two-thirds of patients, the scimitar vein drains the entire right lung, whereas in the remaining one-third it drains only the lower portion of the right lung [2,5,6].

Scimitar syndrome is commonly associated with atrial septal defect (ASD), pulmonary hypoplasia, and patent ductus arteriosus (PDA) [7].

The diagnosis is established by demonstrating partial anomalous pulmonary venous return from the right lung to the IVC or right atrium. This can be achieved using cardiac catheterization, computed tomography (CT), or magnetic resonance imaging (MRI). Noninvasive imaging is particularly useful for defining the abnormal anatomy and planning surgical management [2,3]. Scimitar syndrome is generally managed surgically, especially when the pulmonary-to-systemic flow ratio (Qp:Qs) is greater than 1.5, or when it is associated with other complex cardiac anomalies [7,8].

Here, we report the case of an 8-year-old boy with Scimitar syndrome who underwent chest CT Angiogram, chest X-ray, and echocardiographic evaluation. We highlight the typical imaging features of Scimitar syndrome, its associated complex congenital cardiac anomalies, the clinical presentation, and the implications for management of this rare disorder.

## Case report

An 8-year-old child presented to Nauru Medical Center with cough and failure to thrive. The child was born to a 36-year-old G3P3 mother via normal vaginal delivery after a previous cesarean section. Shortly after birth, the infant developed neonatal cyanosis, which initially resolved but later recurred, resulting in a 3-month admission to the neonatal intensive care unit, where oxygen therapy was required. At the age of 3 months, a CT scan of the chest suggested the presence of only a functioning left lung.

During early childhood, the patient was considered medically fragile and required home oxygen therapy until the age of 2 years. Despite early concerns regarding possible long-term disability, the child achieved all developmental milestones appropriately.

Since the age of 2 years, the patient has remained clinically stable, with no hospital admissions and only 1-2 minor respiratory illnesses per year, which were managed in the outpatient department. The child currently attends regular school and is in Grade 2.

The parents reported a history of recurrent respiratory infections since infancy, occasionally associated with symptoms suggestive of cardiac failure. These episodes required hospitalization on 1 occasion for 1 month, during which the patient was treated conservatively. Before the arrival of a new medical team from GMSS, Nauru Medical Center did not have specialist medical services available.

The parents denied any history of tuberculosis, hepatitis, or other chronic infectious diseases. They also denied any drug or food allergies. There was no history of chills, vomiting, diarrhea, or convulsions.

On physical examination, the child appeared comfortable at rest. Oxygen saturation on room air was 88%, likely reflecting chronic adaptation to long-standing hypoxemia. The most notable finding was digital clubbing involving the toes. The remainder of the physical examination was unremarkable.

Laboratory investigations showed an elevated hemoglobin level of 17.6 g/dL, above the normal range, likely to represent secondary polycythemia due to chronic hypoxemia. Other laboratory parameters were within normal limits, including white blood cell count, red blood cell count, neutrophil count and percentage, lymphocyte ratio, and platelet count. Additional investigations, including liver function tests, renal function tests, and immunoglobulin levels, were also normal.

The patient was referred to the radiology department for further cardiac evaluation and underwent transthoracic echocardiography. Echocardiography demonstrated marked cardiac displacement to the right side hemithorax with preserved normal leftward apex orientation and situs solitus (cardiac dextroposition), along with right ventricular hypertrophy and dilation of the right atrium and IVC (Fig. 1). About 8 mm in size an ASD was also identified, showing a bidirectional shunt more form right to left consistent with advanced pulmonary vascular disease.

**Fig. 1:**
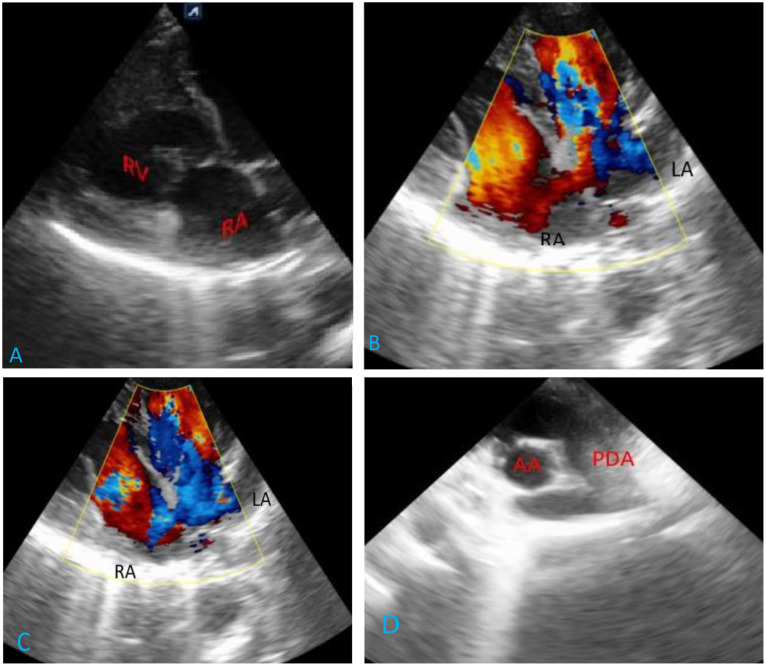
Transthoracic echocardiography showed pronounced right ventricular hypertrophy with dilatation of the right atrium (A). Atrial septal defect (ASD) was identified with bidirectional shunting (B and C), along with a large patent ductus arteriosus (PDA) (D), all in the setting of severe pulmonary hypertension. AA, aorta, LA, left atrium, PDA, patent ductus arteriosus, RA, right atrium, RV, right ventricle.

There was a large PDA and a dilated main pulmonary artery with pulmonary HTN measuring 44 mmHg. The PDA demonstrated bidirectional flow on color Doppler imaging.

The left pulmonary veins drained into the left atrium, but the right pulmonary veins were not visualized draining into the left atrium. Instead, they appeared to course inferiorly and join the IVC, raising strong suspicion for anomalous pulmonary venous return with complex congenital cardiac anomalies and advanced pulmonary hypertension resulting in Eisenmenger syndrome.

A chest radiograph was subsequently performed. This showed a significant reduction in right lung volume with rightward mediastinal shift and relative hyperinflation of the left lung. The cardiac silhouette was difficult to delineate clearly due to the marked dextroposition and right lung hypoplasia (Fig. 2). In view of the suspected complex pulmonary venous anomaly, CT angiography of the chest was obtained for detailed anatomical assessment.

**Fig. 2:**
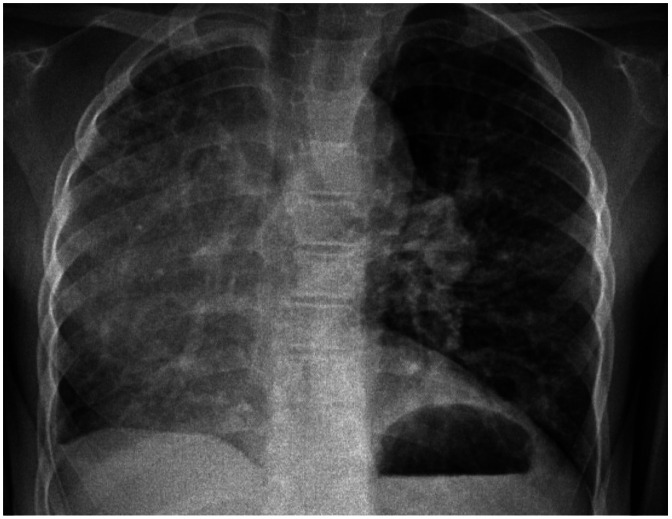
Right lung volume loss is evident, with ipsilateral shift of the mediastinum toward the right side. Multiple linear vascular markings are seen in the right lung, and the left pulmonary artery appears prominent. The cardiac silhouette is poorly visualized, likely due to marked dextroposition and right lung hypoplasia.

CT angiography revealed a severely hypoplastic right pulmonary artery and a markedly enlarged main pulmonary artery, with an increased pulmonary-to-aortic diameter ratio (2.89/1.55 = 1.9) (Fig. 4, Fig. 5), consistent with severe pulmonary hypertension. The right lung was hypoplastic, associated with rightward mediastinal shift and compensatory hypertrophy of the left lung (Fig. 3, Fig. 4). The pulmonary venous anatomy was abnormal: All the right pulmonary veins drained anomalously into the IVC above the diaphragm rather than into the left atrium (Fig. 5). In addition, the right lung received systemic arterial supply from branches arising directly from the abdominal aorta just superior to the celiac trunk, coursing upward to supply predominantly the entire right lung (Fig. 5).

**Fig. 3:**
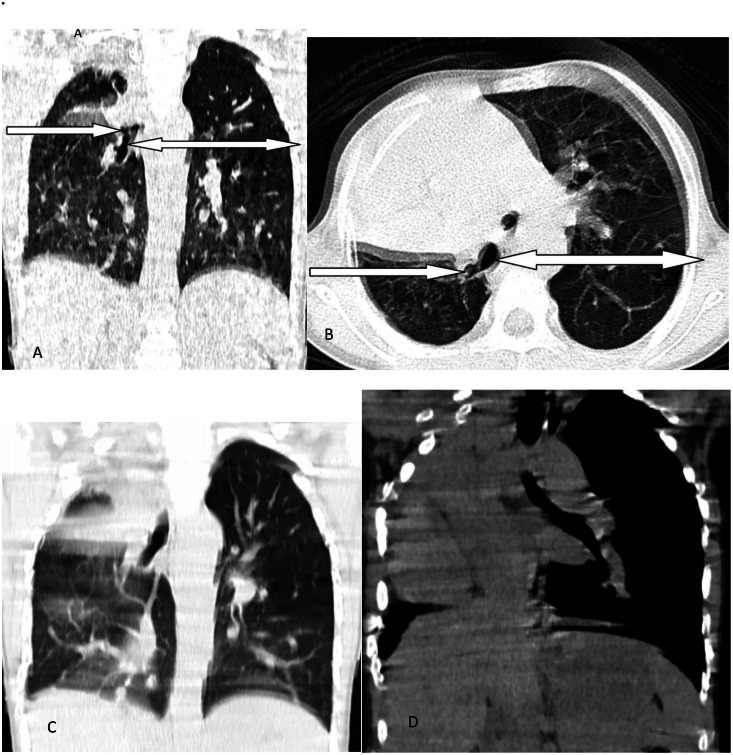
Coronal (A), axial (B), and coronal (C) lung-window images, and coronal (D) soft-tissue-window image demonstrate a hypoplastic right lung with compensatory hypertrophy of the left lung and rightward mediastinal shift, resulting in dextroposition of the heart. A single-head arrow indicates the hypoplastic right upper lobe bronchus, and a double-head arrow indicates the right lower lobe bronchus. No middle lobe bronchus is identified. There is multiple vascular marking in the right lung which converge to single vessel suggestive of abnormal vein (scimitar vein). Single-head arrow (→) = hypoplastic right upper lobe bronchus; double-head arrow (↔) = right lower lobe bronchus.

**Fig. 4:**
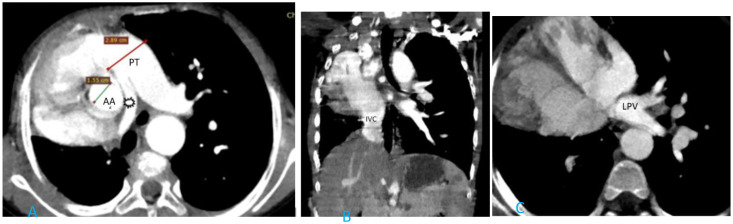
Chest CT angiography demonstrates the following: axial image (A): shows, Dextroposition of the cardiac with a markedly enlarged main pulmonary trunk (PT) and left pulmonary artery. The pulmonary artery–to–aortic diameter ratio (PT/AA) is increased (2.89/1.55 = 1.9), indicating severe pulmonary hypertension. The right main pulmonary artery is severely hypoplastic (burst star). Coronal image (B): shows dilatation of the right atrium and inferior vena cava (IVC) with reflux of contrast into the IVC, suggesting elevated right atrial pressure. Axial image at the level of the pulmonary veins (C): the left pulmonary vein (LPV) drains normally into the left atrium; no right pulmonary vein draining into the left atrium is identified. The right ventricle is hypertrophied. AA, ascending aorta; IVC, inferior vena cava; LPV, left pulmonary vein, PT, pulmonary trunk.

**Fig. 5:**
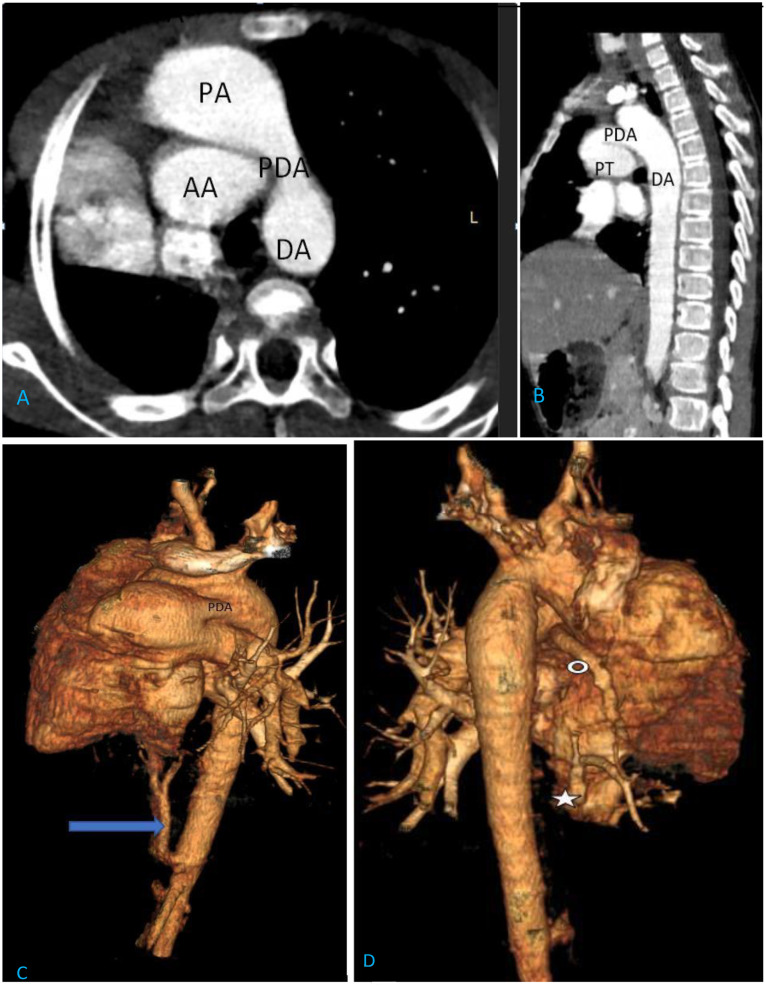
Axial and sagittal contrast-enhanced CT images (A and B) demonstrate a large, short, and wide patent ductus arteriosus (PDA) arising from the descending aorta (DA) and connecting to the main pulmonary artery/pulmonary trunk (PT), with associated dilatation of the pulmonary artery. Three-dimensional volume-rendered CT angiography images (C and D) provide a comprehensive overview of the vascular anomalies. They show: an aberrant systemic artery arising from the abdominal aorta and coursing superiorly to supply the right lung (blue arrow). A markedly hypoplastic right pulmonary artery (circle). The anomalous scimitar vein (star) descending along the right cardiac border and draining into the inferior vena cava. These reconstructions clearly depict the combination of anomalous systemic arterial supply, hypoplastic right pulmonary artery, and anomalous pulmonary venous drainage characteristic of complex scimitar syndrome. AA, ascending aorta; DA, descending aorta; PDA, patent ductus arteriosus; PA/PT, pulmonary artery/pulmonary trunk; blue arrow = aberrant systemic artery from the abdominal aorta; circle = hypoplastic right pulmonary artery; star = scimitar vein.

The CT angiogram also demonstrated a large PDA (Type B, short, and wide configuration), with dilation of the main pulmonary trunk and left pulmonary artery (Fig. 4, Fig. 5). There was marked enlargement of the right atrium and right ventricle with myocardial hypertrophy, reflecting long-standing pressure overload (Fig. 1, Fig. 4). These findings, together with the right-to-left shunt at the atrial level (Fig. 1) and severe pulmonary hypertension and hemoglobin level of 17.6 g/dL and physical finding of differential clubbing, are consistent with Eisenmenger physiology.

Three-dimensional volume-rendered CT angiography images (Fig. 5C and D) provide a comprehensive overview of the vascular anomalies. They show: an aberrant systemic artery arising from the abdominal aorta and coursing superiorly to supply the right lung (blue arrow). A markedly hypoplastic right pulmonary artery (circle). The anomalous scimitar vein (star) descending along the right cardiac border and draining into the inferior vena cava.

Overall, the constellation of findings confirmed the diagnosis of severe, complex Scimitar syndrome with Eisenmenger syndrome, characterized by right lung hypoplasia, anomalous pulmonary venous drainage to the IVC, systemic arterial supply to the right lung, severe pulmonary arterial hypertension, and associated congenital cardiac defects.

Following confirmation of the diagnosis, the patient was managed conservatively with supportive treatment. Given the complexity of the congenital anomalies and the presence of Eisenmenger physiology, definitive management would require specialized multidisciplinary care. After consultation with a pediatric cardiologist and a cardiac surgeon, the decision was made to continue conservative management because of the advanced stage of the disease.

## Case discussion

Scimitar syndrome is a rare variant within the spectrum of congenital cardiovascular anomalies known as partial anomalous pulmonary venous return. Its hallmark is an anomalous right pulmonary vein that drains part or all the right lung into the IVC, right atrium, or portal vein. Associated anomalies are variable and include [1]: hypoplasia of the right lung [2], dextroposition of the heart [9], hypoplasia of the right pulmonary artery (RPA) [3], and anomalous systemic arterial supply from the aorta to the right lung [5,10].

The term “scimitar syndrome” is derived from the characteristic radiographic appearance of the anomalous vein, which manifests as a tubular opacity coursing parallel to the right cardiac border, resembling the curved Turkish sword known as a scimitar [5,11]. This is the classic “scimitar sign.”

All these classic features were present in our patients, with additional abnormalities including a large PDA, large ASD, dilated pulmonary trunk, right ventricular hypertrophy, and right atrial enlargement and Contrast reflux into the IVC indicated elevated right atrial pressure.

A long-standing left-to-right shunt—where oxygenated blood from the lungs is diverted back into the right side of the heart—was present via the scimitar vein draining into the right atrium. This was further augmented by additional left-to-right flow through a PDA and an ASD. Over time, the resulting volume overload and severe pulmonary hypertension led to progressive pulmonary vascular disease and eventual shunt reversal. Clinically, this manifested as chronic hypoxemia (oxygen saturation 88%), digital clubbing, and secondary erythrocytosis with an elevated hemoglobin level (17.6 g/dL). These features are consistent with the development of Eisenmenger syndrome and explain the marked right heart enlargement and persistent hypoxemia observed in this patient.

Scimitar syndrome is a rare congenital anomaly with an estimated incidence of approximately 1-3 per 100,000 live births. The true incidence may be higher, as many older children and adults remain asymptomatic, with some cases detected incidentally on routine chest radiographs [2,5,10].

Patients with the infantile form of Scimitar syndrome are generally diagnosed within the first few months of life. Failure to thrive, tachypnea, and heart failure are the main findings in these severely ill and debilitated infants. Cyanosis may occur if pulmonary hypertension develops, leading to a right-to-left shunt as seen in our patients. Mortality in this group may reach up to 45%. The pulmonary-to-systemic flow ratio (Qp/Qs) is always above 1 unless Eisenmenger physiology has developed [6]. Multiple factors contribute to pulmonary hypertension, including a large left-to-right shunt via the anomalous pulmonary vein, left-to-right shunt from systemic arterial supply to the right lung, right lung hypoplasia with reduction of the pulmonary vascular bed, pulmonary vein stenosis or obstruction, other associated congenital cardiac malformations, and persistent pulmonary hypertension [4,6,11]. In our case left-to-right shunt via the anomalous pulmonary vein, left-to-right shunt from systemic arterial supply to the right lung, right lung hypoplasia with reduction of the pulmonary vascular bed, pulmonary vein stenosis, congenital cardiac malformations (ASD and PDA) and pulmonary hypertension are present.

The adult form of Scimitar syndrome is typically characterized by mild symptoms and a favorable prognosis. In contrast, the infantile form, which presents symptomatically shortly after birth, is frequently complicated by pulmonary hypertension and heart failure, resulting in a poor prognosis [3,5,12].

Our case initially presented cardiac failure during infancy and required a 3-month hospital admission. Referral abroad was not possible at that time because the patient was unable to tolerate air travel. In addition, multidisciplinary specialty services were not available, and the diagnosis of Scimitar syndrome was likely missed during the initial evaluation. The absence of early recognition and timely referral resulted in delayed management. Consequently, the patient later developed irreversible pulmonary hypertension with Eisenmenger physiology. Early diagnosis and appropriate referral to a specialized center might have allowed timely intervention and potentially prevented progression to advanced pulmonary vascular disease.

The severity and clinical manifestations of Scimitar syndrome are largely influenced by the age at diagnosis and the presence of associated congenital cardiac anomalies [8]. In cases of partial anomalous pulmonary venous return draining into a systemic vein or the right atrium, a left-to-right shunt is created. This shunt is often well tolerated and may remain clinically silent; however, symptoms are more likely to develop when the pulmonary-to-systemic flow ratio (Qp:Qs) equals or exceeds 2:1 [2,13].

In two-thirds of patients, the scimitar vein drains the entire right lung; in the remaining one-third, it drains only the lower portion of the right lung.

In a case series of 34 patients with Scimitar syndrome [8]:

Twenty-five patients (73.5%) had both the right superior pulmonary vein (RSPV) and right inferior pulmonary vein (RIPV) converging and draining into the IVC.

Two patients (5.9%) had mixed drainage, with the RSPV draining into the IVC and the RIPV draining into the right atrium (RA).

Four patients (11.7%) had either the RSPV or RIPV draining separately into the IVC.

Two patients (5.9%) had the RSPV and partial branches of the RIPV draining into the IVC.

One patient (2.9%) demonstrated a rare variant in which the right pulmonary vein descended via a vertical vein, connected to the portal vein, and ultimately drained into the IVC through a hepatic vein.

In our case, all right upper and right lower lobe pulmonary veins converged and drained into the supradiaphragmatic IVC. The main anomalous pulmonary vein had a straight orientation with stenosis at the IVC–pulmonary vein junction. The scimitar vein distant from the left atrium (Fig. 5, Fig. 6) which is important for surgical intervention criteria.

**Fig. 6:**
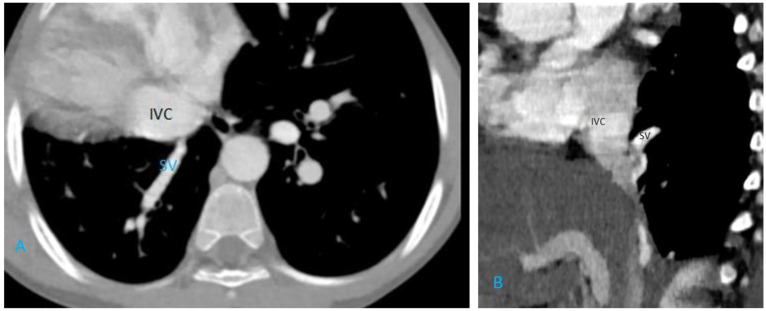
Axial (A) and sagittal (B) postcontrast chest CT images demonstrate a horizontally oriented anomalous right pulmonary vein consistent with a scimitar vein (SV), coursing inferiorly along the right cardiac border and draining into the supradiaphragmatic inferior vena cava (IVC). There is a focal segment of luminal narrowing at the junction of the scimitar vein and the IVC, suggesting venous stenosis at the drainage site. The adjacent IVC is opacified with contrast, confirming the site of entry of the scimitar vein. These features are characteristic of Scimitar syndrome and correlate with anomalous right pulmonary venous return and potential outflow obstruction, which may contribute to elevated right-sided pressures and pulmonary hypertension. IVC, inferior vena cava; SV, scimitar vein.

Pulmonary hypoplasia in Scimitar syndrome is characterized by incomplete development of 1 or more lobes, most frequently involving the upper and middle lobes of the right lung. The right main bronchus is typically bilobed and hyparterial, mirroring the morphology of the left lung (i.e., left isomerism). These pulmonary anomalies are critical for differentiating Scimitar syndrome from uncomplicated pulmonary hypoplasia or isolated partial anomalous pulmonary venous connection.

The primary radiologic distinction between Scimitar syndrome and isolated pulmonary hypoplasia lies in the presence of anomalous pulmonary venous drainage (the “scimitar vein”) and systemic arterial collaterals, both of which are entirely absent in isolated lung hypoplasia. While both conditions share structural characteristics like a small hemithorax and mediastinal shift, cross-sectional imaging easily differentiates them by assessing the vascular anatomy.

The hypoplastic lung may derive systemic arterial supply directly from the aorta or celiac axis in scimitar syndrome. These aberrant vessels usually enter via the inferior pulmonary ligament and represent persistent embryonic aortic postbranchial arches that vascularized the lung buds prior to maturation of the main pulmonary artery [10,13].

Pulmonary artery hypoplasia can be mild, as in most cases, or severe to the point of near absence. The degree of pulmonary artery hypoplasia determines the extent of systemic arterial supply to the hypoplastic lung. In our case, the right lung was bilobed with a hypoplastic right upper lobe (Fig. 3). The right pulmonary artery was very hypoplastic (Fig. 4, Fig. 5), with large systemic arterial supply to the right lung from a branch of the abdominal aorta, coursing upward to supply predominantly the entire right lung (Fig. 5).

Review of the literature shows that right ventricular enlargement is the most common radiological finding associated with Scimitar syndrome, and ASD is the most frequent concomitant congenital heart disease [9,8,14]. Noncardiac anomalies that may be associated with Scimitar syndrome include pulmonary sequestration (commonly of the extralobar type), horseshoe lung, ipsilateral diaphragmatic anomalies, genitourinary anomalies, and vertebral anomalies.

A case report from Ethiopia described a 3-day-old male neonate with imperforate anus presenting with abdominal distention. Chest X-ray showed a scimitar sign, and abdominal ultrasound, transthoracic echocardiography, and CTA confirmed a scimitar vein with other cardiopulmonary malformations. At that time, only 4 cases of coexisting imperforate anus and Scimitar syndrome had been reported worldwide [15].

Another study described a unique bilateral Scimitar syndrome case in an infant with involvement of both lungs, totally anomalous pulmonary venous connection, horseshoe lung, and a right pulmonary cyst [16]. To date, only about 10 such bilateral cases have been reported worldwide.

Scimitar syndrome should be suspected in patients with respiratory distress and/or heart failure in association with radiological findings of cardiac dextroposition and right lung haziness or suspected atelectasis. The dextroposition of the cardiac silhouette may sometimes obscure the scimitar vein, which is often the only clue on a frontal chest radiograph, as in our patient. Contrast-enhanced chest CT is valuable for confirming the diagnosis and identifying associated anomalies.

Chest radiography may provide the first clue to the diagnosis of Scimitar syndrome; however, the initial diagnostic evaluation is typically performed with echocardiography and subsequently confirmed using advanced imaging modalities such as CT or MRI [3]. Although conventional catheter-based angiography has historically been regarded as the gold standard for evaluating the anomalous scimitar vein, it is an invasive procedure.

Given that Scimitar syndrome represents a complex form of anomalous pulmonary venous return, multidetector CT and MRI now enable accurate, noninvasive diagnosis. Both CT and MRI play a crucial role in preoperative assessment by identifying patients suitable for surgical intervention and facilitating operative planning. While precise anatomical delineation is the primary objective, MRI additionally quantifies the pulmonary-to-systemic flow ratio (Qp/Qs), thereby providing functional assessment of shunt severity. Phase-contrast cine MRI allows quantification of pulmonary venous blood flow and differential flow to the right and left lungs [6,13,17].

Pulmonary hypertension in infants with this condition can occur due to several mechanisms, such as decreased right-sided pulmonary vascular bed, scimitar vein stenosis, unrecognized systemic collateral blood flow, associated intracardiac shunts, or other comorbidities such as abnormal lung development [5,11]. It can also be complicated by Eisenmenger syndrome if the diagnosis is delayed and no appropriate treatment is undertaken. Early diagnosis and timely repair of congenital heart disease have led to a decline in Eisenmenger syndrome associated with simple defects in high-income countries. However, Eisenmenger physiology still occurs in patients with complex cardiac anatomy or unrepaired shunt lesions, particularly in low- and middle-income countries, where it often presents later in the course of disease and may benefit from advanced medical therapy [18]. Only 2 case reports of Eisenmenger syndrome related to Scimitar syndrome managed nonsurgically with medical therapy have been published in the English literature [8]. Our case represent the third case Eisenmenger syndrome related to Scimitar syndrome which is managed conservatively because of the late diagnosis.

Asymptomatic adults or older children with Scimitar syndrome and only small shunts can lead normal lives without surgical intervention; they require only regular follow-up [2]. Surgery is the definitive treatment for symptomatic Scimitar syndrome, most often in the infantile form. The surgical approach to correction varies according to the specific anatomy and surgeon preference [8]. Surgical treatment should be considered in patients with hemodynamically significant left-to-right shunting (Qp:Qs greater than 2:1), evidence of right heart failure, recurrent pulmonary infections, or in those undergoing repair of other major cardiac lesions [2,13,17]. Our case had complex Scimitar syndrome complicated by Eisenmenger syndrome and was treated medically after consultation with a pediatric cardiovascular surgeon and a cardiologist because the disease was at an advanced, inoperable stage.

## Conclusion

Scimitar syndrome is a rare cardiopulmonary malformation. Its diagnosis requires a multidisciplinary approach and the use of multiple imaging modalities [5,8]. Symptoms and timing of presentation depend on the severity and combination of associated cardiac and vascular anomalies, with most cases presenting in infancy or early childhood. Surgical treatment mainly aims to redirect the pulmonary venous return to the left atrium and may require lobectomy in selected cases. For older children and adults who are asymptomatic with small shunts, a strategy of regular follow-up is appropriate [2,8]. Prognosis depends on the severity of vascular lesions and associated anomalies. Prognosis is particularly guarded in infants who present early with severe disease; in such settings, multimodality imaging and early referral are critical, especially in resource-limited environments.

## Teaching points

The diagnosis of Scimitar syndrome is predominantly imaging-based. Early recognition by radiologists—using chest radiography, echocardiography, CT, and MRI—can facilitate timely referral and intervention, reduce long-term complications, and improve patient outcomes.

## Data availability

The authors confirm that the data supporting the findings of this study are available within the article and its supplementary materials.

## Author contributions

**Berihu Tadishu Gebre:** Writing—review and editing. Writing—original draft, visualization, conceptualization. **Yirgalem Gereziher:** review and editing, visualization, conceptualization. **Catherine Alcober:** Investigation, conceptualization. **Gladys Baltazar:** Investigation, conceptualization. Viachaslau Markau: Investigation, conceptualization.

## Patient consent

Written informed consent was obtained from the patient’s mother for publication of this case report and accompanying images. A copy of the written consent is available for review by the Editor-in-Chief of this Journal on request.
